# Comparison of perioperative outcomes with or without renorrhaphy during open partial nephrectomy: A propensity score-matched analysis

**DOI:** 10.1590/S1677-5538.IBJU.2016.0581

**Published:** 2018

**Authors:** Hidekazu Tachibana, Toshio Takagi, Tsunenori Kondo, Hideki Ishida, Kazunari Tanabe

**Affiliations:** 1 Department of Urology, Tokyo Women's Medical University, Tokyo, Japan

**Keywords:** Kidney, Carcinoma, Renal Cell, Nephrectomy

## Abstract

**Purpose:**

Renorrhaphy in partial nephrectomy may damage intraparenchymal vessels and compress the renal parenchyma, which may lead to the formation of renal artery pseudoaneurysms or vascularized parenchymal volume reduction. Using propensity score matching, we compared surgical outcomes following non-renorrhaphy and renorrhaphy techniques for open partial nephrectomy (OPN) for T1a renal tumors.

**Materials and Methods:**

We retrospectively analyzed data from 159 patients with normal contralateral kidneys who underwent OPN for T1a renal tumors and pre- and postoperative enhanced computed tomography between 2012 and 2015. Patient variables were adjusted using 1:1 propensity score matching between the two Groups: renorrhaphy (inner and outer layer sutures) and non-renorrhaphy (inner layer sutures only). Postoperative complications and renal function were compared between the two groups.

**Results:**

We matched 43 patients per Group. Operative time, estimated blood loss, cold ischemic time, and postoperative hospital stay were not significantly different between the two Groups. Urine leakage (Clavien-Dindo grade ≥3) occurred in 0 renorrhaphy cases and 2 non-renorrhaphy cases (0% versus 4.6%, P=0.49). Renal artery pseudoaneurysm (RAP) occurred in 6 renorrhaphy cases and in 0 non-renorrhaphy cases (13% versus 0%, P=0.02).

**Conclusions:**

The non-renorrhaphy technique may result in a lower risk of RAP but a greater risk of urine leakage. This technique needs further refinement to become a standard procedure for OPN.

## INTRODUCTION

Partial nephrectomy is the standard of care for small renal masses. In terms of oncological outcomes, partial nephrectomy is comparable to radical nephrectomy for such tumors. Partial nephrectomy is recommended to preserve renal function, which leads to the prevention of end-stage renal disease and cardiovascular events, and subsequently, the extension of patient survival (1-3). To prevent postoperative loss of renal function, a short ischemia time and the preservation of effective renal parenchyma are important. During surgery, many surgeons have contrived new techniques to reduce warm ischemia time, such as the early unclamping technique, zero ischemia technique, and selective renal artery clamping (4-6). To preserve the effective renal parenchyma, minimal margin resection and minimal renorrhaphy are necessary because the renal parenchyma will be compressed by the sutures; in addition, renal blood flow will be reduced. In order to minimize the influence on renal function, various techniques, including the sliding clip technique, V-hilar suture, and non-bolstered horizontal mattress suturing, have been presented (7-10). However, with blind parenchymal suturing, intraparenchymal vessels may be injured, which may cause renal artery pseudoaneurysm (RAP) after surgery (11). At our institution, we have used a non-renorrhaphy technique for open partial nephrectomy (OPN) procedures since 2012 based on the idea that it preserves the effective renal parenchyma and prevents postoperative RAP. In the present study, we aimed to evaluate the preservation rate of renal functional outcomes and the postoperative complication rate of the non-renorrhaphy technique in OPN.

## MATERIALS AND METHODS

Institutional review board approval was obtained to retrospectively analyze patient data. A total of 167 patients underwent OPN for T1a renal tumors at a single institution between 2012 and 2015. The surgeries were primarily performed by three experienced surgeons. Of these cases, patients whose follow-up time and outcome data were available were included in the study. Patients who had an anatomically or functionally solitary kidney or those who could not undergo enhanced computed tomography (CT) because of allergic reaction or impaired renal function were excluded.

In 2012, we elected to carry out an institutional review board-approved CT study after partial nephrectomy for all patients who had no relative or absolute contraindications to the administration of intravenous contrast. In total, 158 patients were eligible for the study. The following variables were considered for each patient: age, sex, height, body weight, presence of hypertension (HT) or diabetes mellitus (DM), tumor diameter, RENAL nephrometry score, pre-and postoperative renal function, operating time, estimated blood loss, cold ischemia time (CIT), and incidence of complications, including RAP and urine leakage. Renal function was assessed using the estimated glomerular filtration rate (eGFR) before and 3 months after surgery. eGFR was calculated using the Modification of Diet in Renal Disease 2 equation (MDRD2) modified for Japanese patients as outlined by The Japanese Society of Nephrology (eGFR=1.94×serum creatinine mg/ dL^1.094^×age×0.739 [if female]) (12).

**Figure 1:**
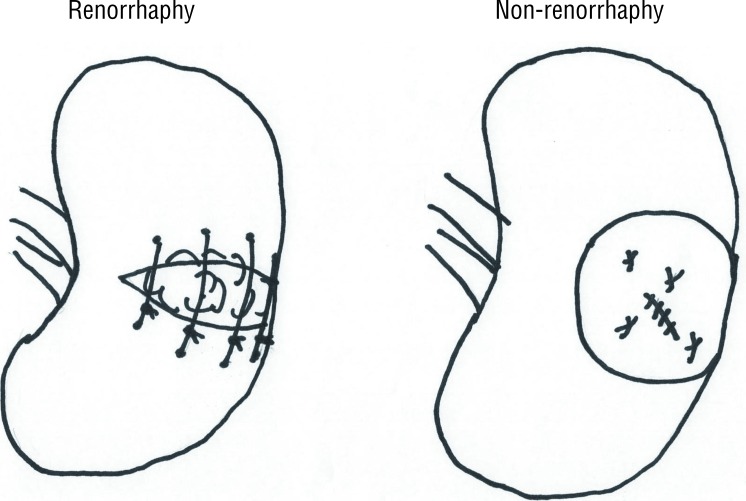
The illustration of the renorrhaphy and non-renorrhaphy technique.

In the renorrhaphy technique, a retroperitoneal approach was made with flank incision. The renal artery and vein were clamped en bloc at the renal hilum. After 5 minutes of cooling with ice slush, the tumor was resected with 2–5 mm of the parenchymal margin; the transected vessels were ligated with 4-0 absorbable sutures and the opened collecting system was repaired with 4-0 absorbable sutures. The renal parenchyma was coagulated with monopolar coagulation (SOFT COAG, VIO 300D; ERBE Elektromedizin, Tubingen, Germany), avoiding the renal hilum, and parenchymal repair was performed with blind 1-0 absorbable interrupted sutures (1-2cm pitch and width) with oxidized cellulose (Surgicel; Ethicon Inc., Somerville, NJ, USA) stuffed in the parenchymal defect. In the non-renorrhaphy technique, parenchymal repair with blind 1-0 absorbable interrupted sutures was omitted; a Tachosil tissue-sealing sheet (CSL Behring Japan, Tokyo, Japan) was placed in the resected bed and manually compressed for 5 minutes after unclamping the renal hilum (Figure-1). To simplify the procedure, we do not routinely use intraoperative indigotindisulfonate injection through an ipsilateral ureteral catheter. If there was no urine leak or RAP upon contrast-enhanced CT angiography (CTA) on postoperative day 3, the drain was removed and patients were discharged. Urine leakage was defined as persistent drain output more than 48 hours after partial nephrectomy with a chemical analysis consistent with urine (13).

To minimize selection bias between patients who underwent renorrhaphy or non-renorrhaphy, patient variables, including age, sex, height, body weight, presence of HT or DM, tumor diameter, RENAL nephrometry score (4–12), and preoperative eGFR, were adjusted using 1:1 propensity score matching. Postoperative outcomes were then compared between patients with and without renorrhaphy in OPN. All statistical analyses were done with JMP 11 (SAS Institute, Cary, NC, USA). Propensity scores were calculated using a multivariable logistic regression model by nearest-neighbor matching with a caliber of 0.2. The Student t test was used for continuous variables. The chi-squared test was used to estimate unordered categorical variables, and the Mann-Whitney U test was used to adjust for ordinal categorical variables.

P < 0.05 was considered statistically significant.

## RESULTS

Table-1 lists pre-and post-matching patient characteristics. Forty-nine patients underwent OPN with renorrhaphy and 109 patients underwent OPN with non-renorrhaphy. Height, body weight, and presence of HT were significantly different between the two Groups. After adjusting for patient variables using 1:1 propensity score matching, we matched 43 patients per Group. After matching, the mean age was 58.6 (range, 27-82) years mean tumor size 28.9 (range, 10-40) mm and the mean preoperative eGFR was 64 (standard deviation (SD) ±19) mL/min/1.73m^2^. The RENAL nephrometry score distribution of the entire matched cohort was as follows: low complexity (score 4-6), 19 (22%); intermediate complexity (score 7-9), 52 (60%); and high complexity (score 10-12), 15 (17%).

**Table 1:** Characteristics of patients undergoing open partial nephrectomy pre- and post-matching.

	Pre-matching			Post-matching		
	Renorrhaphy	Non-renorrhaphy	P-value	Renorrhaphy	Non-renorrhaphy	P-value
No. of cases	49	109		43	43	
Age, years (range)	57 (31-80)	58 (27-82)	0.75	59 (33-80)	59 (27-82)	0.96
Sex (male/female)	34/15	73/36	0.85	30/13	32/11	0.63
Height, cm	166 (±7.3)	164 (±9.0)	0.03	166 (±7.4)	166 (±8.3)	0.91
Body weight, kg	67 (±12)	63 (±12)	0.04	65 (±11)	68 (±12)	0.81
No. of HT (%)	30 (61)	43 (39)	0.01	24 (55)	24 (55)	1.0
No. of DM (%)	12 (24)	22 (20)	0.53	10 (23)	8 (18)	0.59
Tumor size, mm (range)	28 (8-39)	30 (12-40)	0.30	28 (10-40)	29 (12-40)	0.84
Preoperative eGFR, mL/min/1.73 m^2^	66 (±17)	65 (±19)	0.91	64 (±17)	63 (±18)	0.73
Nephrometry score median (IQR)	8 (6-9)	9 (7-9)	0.12	8 (7-9)	8 (7-9)	0.84
R	1 (1-1)	1 (1-1)	0.19	1 (1-1)	1 (1-1)	0.56
E	2 (1-3)	2 (2-3)	0.55	2 (1-3)	2 (2-3)	0.76
N	2 (2-3)	2 (2-3)	0.22	3 (2-3)	3 (2-3)	0.87
A a/x/p	18/22/9	37/45/27	0.88	16/18/9	15/17/11	0.65
L	2 (1-3)	3 (2-3)	0.23	3 (2-3)	3 (2-3)	0.89

Data are presented as mean (±standard deviation)

**IQR** = interquartile range; **HT** = hypertension; **DM** = diabetes mellitus; **eGFR** = estimated glomerular filtration rate; **R** = radius; **E** = exophytic/endophytic properties; **N** = nearness of the tumor to the collecting system or sinus; **A** = anterior/neither/posterior; **L** = location relative to the polar lines

As listed in Table-2, there was no significant difference in mean operative time, estimated blood loss, CIT, or length of postoperative hospital stay between the matched Groups. As a postoperative complication, we compared the incidence of urine leakage and RAP. Urine leakage occurred in nine non-renorrhaphy cases and in one renorrhaphy case (20% [9/43] versus 2.3% [1/43], respectively, P=0.02), although there was no significant difference in the incidence of Clavien-Dindo grade ≥3 urine leakage between non-renorrhaphy and renorrhaphy cases (4.6% versus 0%, P=0.49). On the other hand, RAP occurred in six renorrhaphy cases but in zero non-renorrhaphy cases (13% [6/43] versus 0% [0/43], respectively, P=0.02).

**Table 2:** Comparison of surgical outcomes.

	Post-matching		
	Renorrhaphy (n=43)	Non-renorrhaphy (n=43)	P-value
Operative time, min	194 (±43)	191 (±39)	0.72
Estimated blood loss, mL	175 (±282)	143 (±205)	0.54
Cold ischemia time, min	43 (±19)	38 (±18)	0.69
No. of positive surgical margin	0	1 (2.3%)	0.47
No. of urine leaks (%)			
All	1 (2.3)	9 (20)	0.02
Clavien grade ≥ 3	0	2 (4.6)	0.49
No. of asymptomatic RAP (%)	6 (13)	0 (0)	0.02
Postoperative hospital stay, days	7.0 (±3.3)	6.0 (±2.4)	0.15
Postoperative eGFR (3 months later)	56 (±19)	61 (±19)	0.23
Postoperative decrease rate in eGFR, %	-12 (±20)	-2.2 (±13)	0.008

Data are shown as mean (±standard deviation); **RAP** = renal artery pseudoaneurysm; **eGFR** = estimated glomerular filtration rate.

In the pre-matched cohorts, 19 patients developed urinary fistula (one with renorrhaphy and 18 without renorrhaphy); one patient without renorrhaphy required transcutaneous drainage 5 weeks after surgery, six patients required ureteral stent drainage, and the other 12 patients did not require additional treatment.

RAP was detected using contrast-enhanced CTA 3 days after surgery; all asymptomatic RAP cases were prophylactically treated with transcatheter arterial embolization (TAE) according to another ongoing clinical study protocol, and none of the cases presented postoperative bleeding. Three months after surgery, there was no significant difference in postoperative eGFR (mL/min/1.73m^2^) between the two Groups (56 versus 61, P= 0.23), but the decreasing rate of eGFR was significantly less in the non-renorrhaphy Group than in the renorrhaphy Group (-12%versus -2.2%, P=0.008).

## DISCUSSION

The results of the present study comparing the incidence of complications demonstrate that the non-renorrhaphy technique in OPN resulted in a lower incidence of asymptomatic RAP and a higher incidence of urinary leakage, although the incidence of Clavien-Dindo grade ≥3 urine leakage did not differ significantly. At our institution, we adopted the non-renorrhaphy technique to reduce the occurrence of postoperative complications, including bleeding, hematoma, and RAP based on the hypothesis that blind large needle outer sutures used in the renorrhaphy technique could damage the intraparenchymal vessels. Actually, our results show that the incidence of vascular complication was higher in the renorrhaphy technique.

Renorrhaphy during OPN is considered one of the challenging parts of the procedure, as it requires tension to be applied with careful consideration of force and angle. Endress et al. studied the optimal tension of the parenchymal suture using fresh porcine kidneys and found that tension causing suture failure was only slightly higher than the tension typically applied during partial nephrectomy, and the acute angles of entry or exit are also likely to cause suture failure (14, 15).

Few reports detailing non-renorrhaphy techniques have been published. Ota et al. reported an initial series of 39 OPNs performed without renorrhaphy. Their outcomes were good, with preservation of postoperative renal function and prevention of urological complications. We began using a non-renorrhaphy technique for OPN in 2012, and have already completed 181 cases. We have already reported the surgical outcomes of non-renorrhaphy cases for T1b renal tumors (11). Forty-one patients underwent OPN without renorrhaphy for tumors >T1b. We evaluated renal function using eGFR and performed a volumetric analysis of vascularized parenchyma before and after the surgery; there was no benefit in terms of preservation of vascularized parenchymal mass for the operated kidney and eGFR compared to that in 50 patients who had undergone renorrhaphy. Regarding postoperative complications, the incidence of urinary fistula tended to be higher, but there was no significant difference between the two Groups. In the present study, the percent decrease in eGFR was higher in the renorrhaphy Group. However, this study did not include renal scintigraphy of the operated kidney and so may not be sufficient to conclude the superiority of the non-renorrhaphy technique for preserving renal function (11).

RAP is a life-threatening complication after partial nephrectomy, and the incidence of delayed hemorrhage from RAP after surgery is reported to range from 1.2 to 7.5% (16, 17). According to the institutional protocol and the approval of the institutional review board, we have routinely performed contrast-enhanced CTA for the screening of asymptomatic RAP in the early postoperative period, and have performed TAE for the prevention of delayed hemorrhage. In our previous study on the incidence of RAP after partial nephrectomy between January 2012 and May 2014, out of 212 cases in which postoperative CTA was not performed, delayed hemorrhage occurred in 10 (4.7%) cases. On the other hand, asymptomatic RAP was detected in 46 (15%) out of 312 cases, which was higher than the rate previously reported for symptomatic RAP. Prophylactic TAE was performed in 26 cases (8%) if the diameter of RAP was >5mm. As a result, delayed hemorrhage occurred in 2 cases (0.6%). We could reduce the incidence of delayed hemorrhage by performing prophylactic TAE for asymptomatic RAP (P=0.005) (18).

The mechanism of RAP development after partial nephrectomy has not been identified, but some possible causes have been reported, such as inadequate hemostasis of arterial bleeding from the resected bed, or injury of the renal vessels due to suturing with large needles in renorrhaphy (19-21). We previously reported that the risk of developing RAP was associated with the renal sinus exposure of renal tumors (22). Blind suture of the parenchyma with large needles has a higher potential to damage large vessels, especially when sutures are placed close to the renal sinus where the segmental arteries run. In the present study, we showed a lower incidence of asymptomatic RAP when using the non-renorrhaphy technique, which is consistent with this hypothesis. There are some concerns about the use of CTA in the early postoperative period for screening because of the adverse effects of contrast materials on renal function and because of radiation exposure. We have already published 4 papers regarding the early use of CTA, and have discussed how the advantages of the early detection of RAP to prevent rupture outweigh these safety concerns (18, 21-23). Our previous study also showed no significant difference in the decrease of eGFR with or without CTA in the early postoperative period (18, 21). Additional radiation exposure may increase the risk of secondary malignancy, but RAP rupture can make patients hemodynamically unstable and require emergent TAE and management in intensive care units. Comparing these risks, prioritization of the risk reduction by detecting asymptomatic RAP might offer greater advantages.

The incidence of urine leakage in OPN is reported to range from 1.0 to 17.4%; this incidence is higher than that in minimally invasive partial nephrectomy because of the indication for relatively hilar-located tumors in OPN (24). Urine leakage occurs if the collecting system opens during the resection of hilar tumors and is not fully repaired. In our study, the incidence of urine leakage was higher for patients undergoing non-renorrhaphy because of the lack of parenchymal packing with renorrhaphy. When using the renorrhaphy technique even a small collecting system opening, if left unrepaired, would be packed with renal parenchyma. In the present study, the incidence of urine leakage requiring additional treatment (Clavien-Dindo grade ≥3) was not significant. This result might be due to the detection of a small amount of urine leakage, which might have healed spontaneously due to the routine early postoperative CTA at our institution. Therefore, we assume that the differences in the incidence of urine leakage were not clinically important.

This study has several limitations, including its retrospective nature, collection of data from a single institution, and small sample size. The surgical procedure, whether renorrhaphy or non-renorrhaphy, may change depending on the surgeon's intraoperative findings. Patients in the early years of this study underwent renorrhaphy more frequently, whereas those in the later years of the study underwent non-renorrhaphy more often. Moreover, we did not specifically evaluate the operated kidney's function using nuclear renal scans or a volumetric analysis. We evaluated postoperative renal function using global renal function (eGFR) and included only patients with normal contralateral kidneys. The inferiority of postoperative renal function in the renorrhaphy group compared to that in the non-renorrhaphy Group may have been affected by the greater number of TAEs performed because of the greater incidence of asymptomatic RAP in our data set. Therefore, the difference in the percent decrease in eGFR may not be sufficient to conclude the superiority of the non-renorrhaphy technique for preserving renal function. In the present study, patient and tumor characteristics were adjusted using 1:1 propensity score matching and the postoperative complications were compared; asymptomatic RAP was more likely in the renorrhaphy technique and urine leakage was more likely in the non-renorrhaphy technique. Partial nephrectomy is well indicated for T1a renal tumors, so the outcomes of the present study will greatly assist surgeons in deciding whether to use non-renorrhaphy techniques.

In conclusion, for patients with T1a renal tumors, the non-renorrhaphy technique has the advantage of reducing the incidence of asymptomatic RAP, whereas the incidence of urine leakage was higher than that in the renorrhaphy technique. In order to become a standard procedure for OPN, especially for endophytic tumors, surgeons should consider additional techniques to ensure collecting system closure, such as intravenous or ureteral stent intraoperative indigotindisulfonate injection.

## Abbreviations

CIT: = cold ischemia time
CT: = computed tomography
CTA: = computed tomography angiography
DM: = diabetes mellitus
eGFR: = estimated glomerular filtration rate
HT: = hypertension
MDRD: = Modification of Diet in Renal Disease
OPN: = open partial nephrectomy
RAP: = renal artery pseudoaneurysm
TAE: = transarterial embolization
